# Comparing prediction of ongoing pregnancy and live birth outcomes in patients with advanced and younger maternal age patients using KIDScore™ day 5: a large-cohort retrospective study with single vitrified-warmed blastocyst transfer

**DOI:** 10.1186/s12958-021-00767-4

**Published:** 2021-07-02

**Authors:** Keiichi Kato, Satoshi Ueno, Jørgen Berntsen, Motoki Ito, Kiyoe Shimazaki, Kazuo Uchiyama, Tadashi Okimura

**Affiliations:** 1 Kato Ladies Clinic, 7-20-3, Nishi-shinjuku, Shinjuku, Tokyo, 160-0023 Japan; 2 Vitrolife A/S, Jens Juuls Vej, 20 8260 Viby J, Denmark

**Keywords:** Time-lapse incubation, Pregnancy, Minimal ovarian stimulation, Single embryo transfer, Maternal age, Blastocyst culture, Live birth

## Abstract

**Background:**

The KIDScore™ Day 5 (KS-D5) model, version 3, is a general morphokinetic prediction model (Vitrolife, Sweden) for fetal heartbeat prediction after embryo transfer that was developed based on a large data set that included implantation results from a range of clinics with different patient populations, culture conditions and clinical practices. However, there was no study to comparing their pregnancy and live birth prediction ability among different maternal age. The aim of this study is to analyze the performance of KS-D5 in predicting pregnancy and live birth in various maternal age groups after single vitrified-warmed blastocyst transfer (SVBT).

**Methods:**

A total of 2486 single vitrified-warmed blastocyst transfer (SVBT) cycles were analyzed retrospectively. Confirmed fetal heartbeat positive (FHB+) and live birth (LB+) rates were stratified by Society for Assisted Reproductive Technology (SART) maternal age criteria (< 35, 35–37, 38–40, 41–42 and ≥ 43 years of age). Within each age group, the performance of the prediction model was calculated using the AUC, and the results were compared across the age groups.

**Results:**

In all age groups, the FHB+ rates decreased as the KIDScore decreased (*P* <  0.05). Conversely, the AUCs increased as the maternal age increased. The AUC of the < 35 age group (0.589) was significantly lower than the AUCs of the 41–42 age group (0.673) and the ≥43 age group (0.737), respectively (*P* <  0.05). In all age groups, the LB+ rates decreased as the KIDScore decreased (*P* <  0.05). Conversely, the AUCs increased as the maternal age increased. The AUC of the ≥43 age group (0.768) was significantly higher than the AUCs of other age groups (*P* <  0.05; < 35 age group = 0.596, 35–37 age group = 0.640, 38–40 age group = 0.646, 41–42 age group = 0.679).

**Conclusions:**

In the present study, we determined that the KIDScore model worked well for prediction of pregnancy and live birth outcomes in advanced age patients.

**Supplementary Information:**

The online version contains supplementary material available at 10.1186/s12958-021-00767-4.

## Introduction

Single blastocyst transfer is mainly used during in-vitro fertilization (IVF) treatment to prevent multiple conceptions and, it results in higher pregnancy rates than cleavage transfer. When several embryos are available after prolonged embryo culture, a thorough evaluation of each blastocyst is needed to identify those with the highest implantation potential. Since its inception, the blastocyst grading system developed by Gardner et al. based on the degree of blastocyst expansion and the morphology of the inner cell mass and trophectoderm cells has been widely used in various institutions around the world [1]. Furthermore, our previous study suggested that a combination of embryo developmental speed to expanding blastocyst and maternal age could be used to predict pregnancy outcomes [2].

Recently, time-lapse incubation has become available for clinical use. The time-lapse imaging technology has not only enabled embryo culture under stable and uninterrupted conditions, but has also improved the ability to select embryos through consecutive observation [3]. Consecutive observation by means of time-lapse incubation makes it possible to accurately evaluate the developmental speed and the cell cycle duration in an embryo. Embryo developmental parameters such as the timing of pronuclear fading [4], the timing of 2-cell division [5], the timing of 4-cell division, the timing of blastocyst formation [5] and others have been reported to be indicative of pregnancy outcomes. Similarly, typical human embryo cleavage features such as direct cleavage [6], reverse cleavage [7], blastomere movement [8, 9], blastocyst collapse [10, 11] and other morphokinetic events were observed by consecutive observation and were used to predict pregnancy outcomes. The importance of these features was reported by the ESHRE working group [12]. Therefore, morphokinetic parameters are important factors to consider when evaluating embryo quality.

Based on a combination of morphokinetic factors, high-accuracy embryo selection models can be made [5]. For blastocyst culture and transfer, several prediction models for blastocyst selection have been reported predicting implantation [5, 13] or blastocyst formation [14]. However, the performance of these models might differ between different IVF clinics. Therefore, embryo selection models should be validated prior to use [15, 16]. In contrast, the KIDScore™ Day 5 (KS-D5) model, version 3, is a general morphokinetic model for implantation prediction (Vitrolife, Sweden) that was developed based on a large data set from a range of clinics with different patient populations, culture conditions and clinical practices. Thus, KS-D5 is a decision support tool that is intended to be generally applicable in different clinical settings for choosing the blastocyst to transfer.

Although it used the previous version of KS-D5, a recent study published by a French team suggested that KS-D5 could be useful as a support tool for deciding which blastocyst to select for transfer. However, this study did not look into the possible value of pregnancy prediction [17].

Maternal age has a significant influence on pregnancy rates due to the strong correlation with euploidy rates [18, 19]. One study showed that KS-D5 scores to some extent correlated with aneuploidy and were potentially associated with the clinical outcome of single embryo transfers in preimplantation genetic testing for aneuploidy cycles [20]. Furthermore, it has been shown that blastocyst morphology highly correlates with maternal age [21], which may affect KS-D5 scores. Therefore, the use of any blastocyst score in combination with a stratification by maternal age would possibly allow for a more accurate prediction of pregnancy outcomes. Clinically, predicting the actual pregnancy chance would be good for patient communication in order to prevent unrealistic expectations of older patients and to mitigate worries and concerns amongst younger patients. In fact, in our previous study, we established a blastocyst grading system by embryo developmental speed that stratified for maternal age [2]. Therefore, to effectively utilize KS-D5, it is important to investigate the relationship between KS-D5 scores and maternal age.

The aim of the present study was to analyze the performance of KS-D5 in different maternal age groups and to calculate fetal heartbeat positive (FHB+) rates and live birth positive (LB+) rates following single vitrified-warmed blastocyst transfer (SVBT).

## Materials and methods

### Patients and study design

A total of 2486 autologous SVBT cycles were included from 2486 patients that underwent their first SVBT cycle between September 2018 and April 2020. During the study period, only single embryo transfers were performed following an exclusive single embryo transfer policy. Therefore, the cohort was analyzed on a per-cycle basis. Blastocysts for warming were selected based on our internal blastocyst grading model that combines our previous study [2] and the Gardner criteria. The survival rate of thawed blastocysts was 99.8% (2482/2486). Patients who underwent preimplantation genetic diagnosis and women with hypothalamus-pituitary gland-related amenorrhea were excluded. All inseminations were carried out by intracytoplasmic sperm injection (ICSI). The cycles were stratified by Society For Assisted Reproductive Technology (SART) age groups and by KS-D5 score quartiles and analyzed for correlation with FHB. The maternal age and paternal age were used as of egg retrieval (ER). In the first analysis, the AUC for predicting FHB+ or LB+ were compared among maternal age groups. In the second analysis, we showed the estimated coefficients of the logistic regression for calculation of the actual value of FHB+ rates.

The institutional review board approved the study design (approval number: 16–32, approved March 13, 2017). Informed consent was obtained from all couples. They were informed that their anonymized data could be used for retrospective analyses.

### Minimal ovarian stimulation, oocyte retrieval, fertilization procedures and embryo culture

All patients underwent a minimal ovarian stimulation protocol [5]. Ovulation was triggered using buserelin (Suprecur; Mochida Pharmaceutical Co., Ltd., Tokyo, Japan or Buserecur; Fuji Pharma Co., Ltd., Tokyo, Japan), a nasal spray containing a GnRH agonist, after confirmed initiation of the LH surge. ER was performed using a fine 21–22 G needle (Kitazato, Japan) without any anesthesia and without follicular flushing. ICSI was performed when spindle appearance (IX73-SLIMSI, IX83, OLYMPUS, Japan) was confirmed after ER. ICSI was performed 4–5 h after the retrieval. After ICSI, the oocytes were transferred to a pre-equilibrated EmbryoSlide (Vitrolife, Sweden) and incubated in the time-lapse incubator (EmbryoScope+ or EmbryoScope Flex, Vitrolife, Sweden). EmbryoSlides were prepared according to the manufacturer’s instructions. A one-step medium (NAKA medical, Japan) was used for embryo culture. The culture dishes were covered with mineral oil (Ovoil, Vitrolife). All embryos were cultured at 37 °C under a gas phase of 5% O_2_, 6% CO_2_ and 89% N_2_ from day 1–7.

### Embryo observation, blastocyst monitoring, grading and vitrification

Fertilization assessment was performed 16–20 h after ICSI. Normally fertilized zygotes with 2 pronuclei (PN) were cultured until the blastocyst stage. Embryo observation was performed using the EmbryoViewer software (Vitrolife, Sweden) without removing the culture dish from the incubator to confirm the presence of 2 PN on day 1 and embryo cleavage on day 2. As per the center protocol, in order to closely monitor embryonic development between days 5 and 7, embryos were checked twice daily until blastocyst freezing. For blastocyst vitrification on day 5 or 6, the blastocysts were required to attain an inner diameter of > 160 μm [22]. The embryos that fulfilled these blastocyst vitrification criteria were used in our study and were defined as utilized blastocysts. The embryos that we selected in this way were vitrified immediately according to the Cryotop method [23]. If the developing embryo did not fulfill the desired criteria, it was cultured further until a maximum of day 7. For blastocyst vitrification on day 7, the blastocysts were required to attain an inner diameter of > 180 μm [24]. If the embryo did not fulfill this criterion by day 7, it was discarded. Measurements of the blastocyst inner diameter were performed using the EmbryoViewer software. ICM and TE were stratified into three grades (A to C) according to the Gardner criteria [1]. For KS-D5, version 3, only annotations of the timing of pronuclear fading (tPNf), the timing of 2-cell division (t2), the timing of 3-cell division (t3), the timing of 4-cell division (t4), the timing of 5-cell division (t5), the timing of blastocyst formation (tB) and morphological grade (inner cell mass (ICM) and trophectoderm (TE)) according to the Gardner criteria [1] are required. The model calculates a continuous KS-D5 score from 1.0–9.9. Furthermore, this model is limited to embryo culture under reduced oxygen with day 5 transfer. After annotation of the required parameters, KS-D5 scores were calculated using the EmbryoViewer software. The morphological grading and annotations were carried out by well-trained experienced embryologists. Their kappa values for an indication of the consistency of evaluation in blastocyst morphology were 0.6 for ICM and 0.7 for TE. No variations were observed in the morphokinetic, leading to basic parameters consistent with the previous study [25]. Therefore, their consistency in terms of morphokinetics was not analyzed.

### Post-warming embryo culture and vitrified-warmed blastocyst transfer procedure

SVBT was performed on day 4.5–5 after ovulation during a spontaneous natural cycle as previously described [26]. Blastocysts for warming were selected based on our internal blastocyst grading model that combines our previous study [2] and the Gardner criteria. If a patient had more than one blastocyst with similar grades, the blastocyst for transfer was selected based on trophectoderm, ICM, blastocyst diameter and blastocyst expansion time. Therefore, KS-D5 was not used for deciding which blastocyst to transfer. The blastocyst warming was carried out according to Cryotop methods [23]. After warming, laser-assisted hatching (Saturn 5 laser system, Origio, Denmark) was performed for complete zona removal in accordance with our previous study [27]. The blastocysts were cultured for 30 min to 2 h until blastocoel re-expansion was confirmed. Only blastocysts where the blastocoel size remained the same or increased relative to the size before vitrification were transferred. Degenerating blastocysts were discarded.

The embryo transfer procedure was performed by placing a single blastocyst, suspended in a minimal volume of medium, in the upper part of the uterine cavity under vaginal ultrasonographic guidance using a specially designed soft silicone inner catheter (Kitazato, Japan). Luteal support was provided depending on the patient’s serum progesterone (P4) level on the day of the embryo transfer. Patients with a P4 level > 12 ng/mL were administrated dydrogesterone (30 mg/day orally, Daiichi-Sankyo, Japan). SVBT was not carried out on patients with P4 levels < 8 ng/mL. Patients whose P4 levels were in the range of 8 to 12 ng/mL were administrated progesterone intravaginally (Luteum Vaginal Suppository, ASKA Pharmaceutical, Japan) until the eighth week of pregnancy. During the first trimester, pregnancies were followed weekly by performing hormone measurements and ultrasonography until approximately 9 weeks of ongoing gestation (confirmed FHB), at which point patients were referred to their obstetrician for subsequent care. Live birth outcomes were ascertained by a written patient questionnaire and/or by the treating obstetrician.

### Statistical analysis

A chi-squared test was used to compare categorical variables among groups. Nominal variables were analyzed using the Wilcoxon rank-sum test or the Cochran-Armitage test for trend as appropriate. The Pearson correlation coefficient was used to detect linear association. The performance of KS-D5 in predicting FHB was calculated using receiver operating characteristic (ROC) curves. The area under the ROC curves (AUC) that indicated the power of FHB or LB prediction were compared among all maternal age groups using the two-sided DeLong’s test. An AUC of 0.5 is equivalent to random prediction, whereas 1.0 is equivalent to a 100% correct prediction.

Logistic regression was used to analyze the relationship between FHB and KS-D5 scores. Initially, a univariate analysis between FHB and maternal age, paternal age, number of previous egg retrivals, number of previous embryo transfers, cause of infertility and KS-D5 as covariates was performed. The same covariates were used in multi-variable logistic regression. The response to maternal age and KS-D5 was further analysed by including the non-linear quadratic and interactions terms: age*age, KS-D5*KS-D5 and age*KS-D5 as continuous covariate variables in the multi-variable logistic regression [28]. To choose which covariates to include, a stepwise variable selection procedure was used in which variables with the best Akaike information criterion (AIC) were included.

JMP software (version 10.0; SAS Institute, Cary, NC) and R (version 3.6.1, 2019-07-05) were used for all statistical analyses.

## Results

Table 1 shows the participant characteristics for each maternal age group. There were significant differences in the average number of previous egg retrieval cycles and the number of previous embryo transfers between age groups (*P* <  0.05). In terms of etiology of infertility, the < 35 age group has significantly higher rates of ovulation disorders than the ≥43 age group (*P* <  0.05). Furthermore, the < 35 age group has significantly lower rates of unknown factor than the ≥43 age group (*P* <  0.05). Also, the < 35 age group and the 35–37 age group have significantly higher rates of combinations of two or more infertility factors than the ≥43 age group (*P* <  0.05). Additionally, we compared AUCs of KIDScore between male factor infertility and non-male infertility factors. The AUC was 0.678 and 0.683, respectively, and not significant.

**Table 1:** Patients characteristics for each maternal age group

	< 35	35–37	38–40	41–42	≥ 43	Total
Cycle, n	292	405	613	490	682	2482
Maternal age(± SD)at ER	32.1 ± 1.9	36.1 ± 0.8	39.2 ± 0.8	41.5 ± 0.5	44.3 ± 1.4	39.7 ± 4.1
Paternal age(± SD)at ER	35.0 ± 4.9	37.9 ± 4.3	40.6 ± 4.4	43.0 ± 4.6	45.0 ± 5.1	41.2 ± 5.7
No. of previous ER(± SD)	2.2 ± 2.0 ^a^	2.9 ± 2.7 ^b^	3.5 ± 3.5 ^c^	4.5 ± 4.1 ^d^	7.8 ± 7.2 ^e^	4.7 ± 5.2
No. of previous ET(± SD)	1.6 ± 1.6 ^a^	2.3 ± 2.4 ^b^	2.7 ± 2.7 ^c^	3.2 ± 2.9 ^d^	4.3 ± 4.0 ^e^	3.0 ± 3.2
Etiology of infertility (%)						
Male factor	29.7	25.4	25.9	27.5	21.6	25.4
Tubal factor	2.1	5.0	4.5	5.8	5.7	4.9
Endometriosis	5.5	3.8	4.2	2.6	3.7	3.8
Ovulation disorders	4.1 ^a^	3.3 ^ab^	2.2 ^ab^	0.9 ^b^	2.3 ^ab^	2.4
Uterine or cervical factor	3.8	4.0	4.9	6.0	7.0	5.4
Combination	15.5 ^a^	13.6 ^a^	12.4 ^ab^	9.8 ^ab^	8.0 ^b^	11.3
Unknown	39.0 ^a^	44.3 ^ab^	45.8 ^ab^	47.3 ^ab^	51.7 ^b^	46.6
Other	0.3	0.5	0.2	0.2	0.2	0.1

The different letter has statistically significant differences across the maternal age groups (*P* <  0.05)

*ER* Egg retrieval, *ET* Embryo transfer, *SD* Standard deviation, *SE* Standard error

**Fig. 1:**
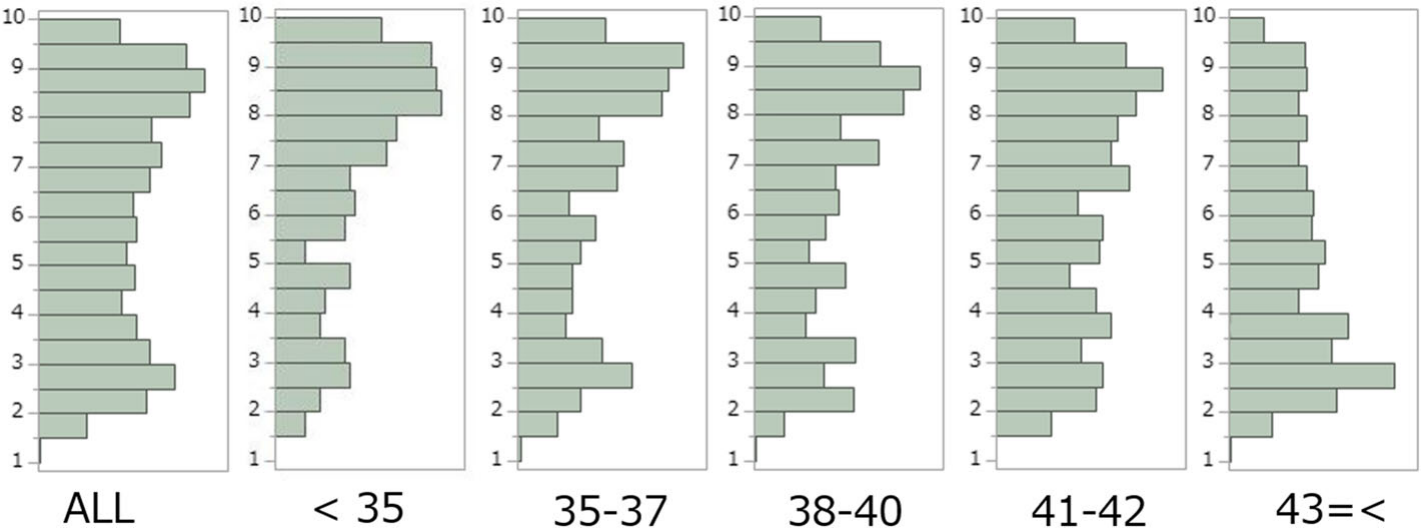
Distribution of KS-D5 scores for all embryos and in different age groups

Table 2 shows the embryo characteristics for each maternal age group. When maternal age increased, tB in general also increased with a significantly shorter tB for the younger groups compared to the older groups (*P* <  0.05). The KS-D5 maximum and minimum scores were not different among maternal age groups. However, Q1, median and Q3 decreased with increased maternal age. There were significant differences in the average KS-D5 scores between age groups (*P* <  0.05), except for adjacent age classes under the age of 43. In the ≥43 age group, we found lower KS-D5 scores than in all other groups (*P* <  0.05). The distribution of KS-D5 scores within each maternal age group is shown in Fig. 1.

**Table 2:** Embryo characteristics for each maternal age group

		< 35	35–37	38–40	41–42	≥ 43	Total
tPNf (hour)		22.8 ± 0.17	22.8 ± 0.16	22.7 ± 0.12	22.7 ± 0.13	22.5 ± 0.12	22.7 ± 0.06
t2 (hour)		25.4 ± 0.18	25.4 ± 0.16	25.2 ± 0.13	25.2 ± 0.13	25.1 ± 0.12	25.2 ± 0.06
t3 (hour)		35.4 ± 0.24	35.7 ± 0.22	35.4 ± 0.18	35.6 ± 0.19	35.4 ± 0.16	35.5 ± 0.09
t4 (hour)		37.0 ± 0.27	37.2 ± 0.23	37.0 ± 0.19	36.9 ± 0.21	37.0 ± 0.18	37.0 ± 0.09
t5 (hour)		48.2 ± 0.42	48.3 ± 0.36	48.3 ± 0.29	48.3 ± 0.32	48.4 ± 0.27	48.3 ± 0.14
tB (hour)		104.7 ± 0.61 ^a^	105.6 ± 0.52 ^ab^	106.2 ± 0.42 ^b^	106.9 ± 0.47 ^b^	108.9 ± 0.40 ^c^	106.8 ± 0.21
Day of cryopreservation (%)	Day 5*	76.7	73.3	71.8	70.0	60.1	69.0
	Day 6*	22.6	25.2	27.1	28.4	37.8	29.5
	Day 7*	0.7	1.5	1.1	1.6	2.1	1.5
ICM grade (%)	A*	58.9	53.3	53.5	50.6	40.3	49.8
	B	24.3	25.4	25.9	28.6	26.7	26.4
	C*	16.8	21.2	20.6	20.8	33.3	23.8
TE grade (%)	A*	55.8	46.2	44.2	39.2	28.3	40.5
	B	20.9	24.2	26.3	25.7	22.7	24.2
	C*	23.3	29.6	29.5	35.1	49.0	35.3
KIDScore	Average	6.68 ± 2.33 ^a^	6.35 ± 2.47 ^ab^	6.23 ± 2.40 ^bc^	6.02 ± 2.39 ^cd^	5.28 ± 2.33 ^e^	6.0 ± 2.4
	MAX	9.8	9.8	9.8	9.8	9.8	9.8
	75%	8.6	8.6	8.4	8.1	7.3	8.2
	Median	7.4	6.9	6.7	6.4	5.1	6.3
	25%	2.9	2.6	2.6	2.6	2.4	3.7
	MIN	1.7	1.4	1.4	1.5	1.3	1.3

Value are mean ± SEM or %

Different subscripts are statistically significant different across the maternal age groups (*P* <  0.05)

* An asterisk means significant correlation between the rates of each grade and maternal age groups by Cochran-Armitage trend test (*P* <  0.05). Timing of pronuclear fading = tPNf); Timing of 2-cell division = t2; Timing of 3-cell division = t3; Timing of 4-cell division = t4; Timing of 5-cell division = t5; Timing of blastocyst formation = tB; *SEM* Standard error of the means, *ICM* Inner cell mass, *TE* Trophectoderm

### FHB+ rates stratified by maternal age group and analysis of KS-D5

Table 3 shows the results of the uni- and multi-variable logistic regression analysis for FHB+. Maternal age and KS-D5 scores correlate with FHB+ (maternal age: adjusted odds ratio (aOR) 0.877, 95% confidence interval (CI) 0.846–0.886; KSs: aOR 1.325, 95% CI 1.270–1.385).

**Table 3:** Uni- and multi-variable logistic regression analysis for FHB+ after SVBT

	Univariate analysis			Multivariate analysis		
	OR ratio	95% CI	*P* value	aOR ratio	95% CI	*P* value
Maternal age	0.866	0.846–0.886	< 0.05	0.877	0.850–0.905	< 0.05
Paternal age	0.944	0.928–0.960	< 0.05	–		0.291
No. of previous ER	0.929	0.906–0.950	< 0.05	–		0.179
No. of previous ET	0.937	0.906–0.968	< 0.05	–		0.558
Cause of infertility	–		0.547	–		
KS-D5 scores	1.325	1.270–1.385	< 0.05	1.282	1.227–1.340	< 0.05

*ER* Egg retrieval, *ET* Embryo transfer, *KS-D5* KIDScore™ Day 5, *SVBT* Single vitrified-warmed blastocyst transfer, *FHB+* Fetal heart beat positive, *OR* Odds ratio, *aOR* Adjusted odds ratio, *CI* Confidence interval

Table 4 shows the FHB+ rates in each KS-D5 group stratified by maternal age. In all age groups, FHB+ rates significantly increased when KS-D5 scores increased (*P* <  0.05). Furthermore, within each maternal age group, FHB+ rates significantly increased when KS-D5 scores increased (*P* <  0.05). In all KS-D5 groups, FHB+ rates significantly decreased when maternal age increased (*P* <  0.05).

**Table 4:** FHB+ rates (%) in each KS-D5 group stratified by SART maternal age

	1.0–3.6*	3.7–6.0*	6.1–8.0*	8.1–9.9*	No. of cycles	*P* value for trend test
< 35	26.5	38.2	41.0	50.0	295	< 0.05
35–37	20.2	31.8	39.4	52.2	405	< 0.05
38–40	13.1	21.2	33.3	38.5	613	< 0.05
41–42	8.6	14.6	23.1	36.4	490	< 0.05
≥ 43	3.1	6.6	12.9	27.7	682	< 0.05
Total	10.7	17.8	28.6	41.0	2482	< 0.05

*FHB+* Fetal heart beat positive, *AUC* Area under the curve for prediction of FHB+, *KS-D5* KIDScore Day5

* An asterisk indicates that the FHB+ rates significantly decreased when maternal age increased for each column according to the Cochran-Armitage trend test (*P* <  0.05)

**Fig. 2:**
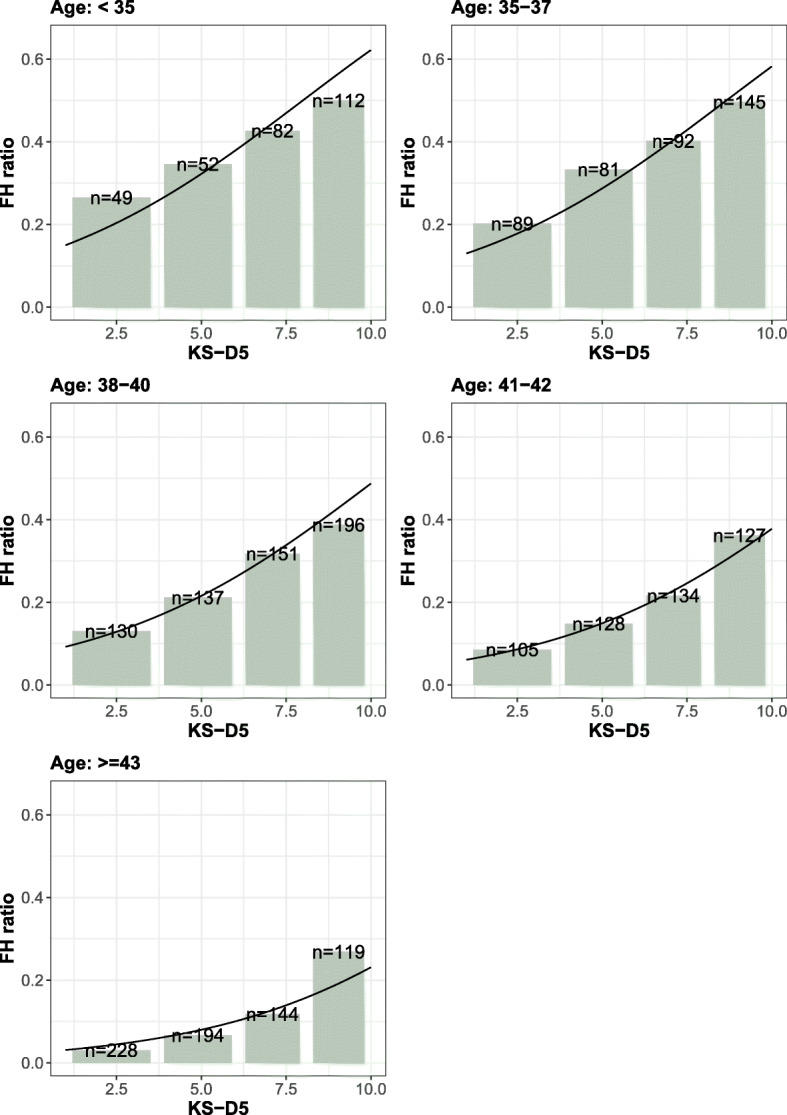
The fetal heartbeat ratio in different KS-D5 score groups. The panels show the response for each maternal age group. The line is the fitted multivariate logistic regression

**Fig. 3:**
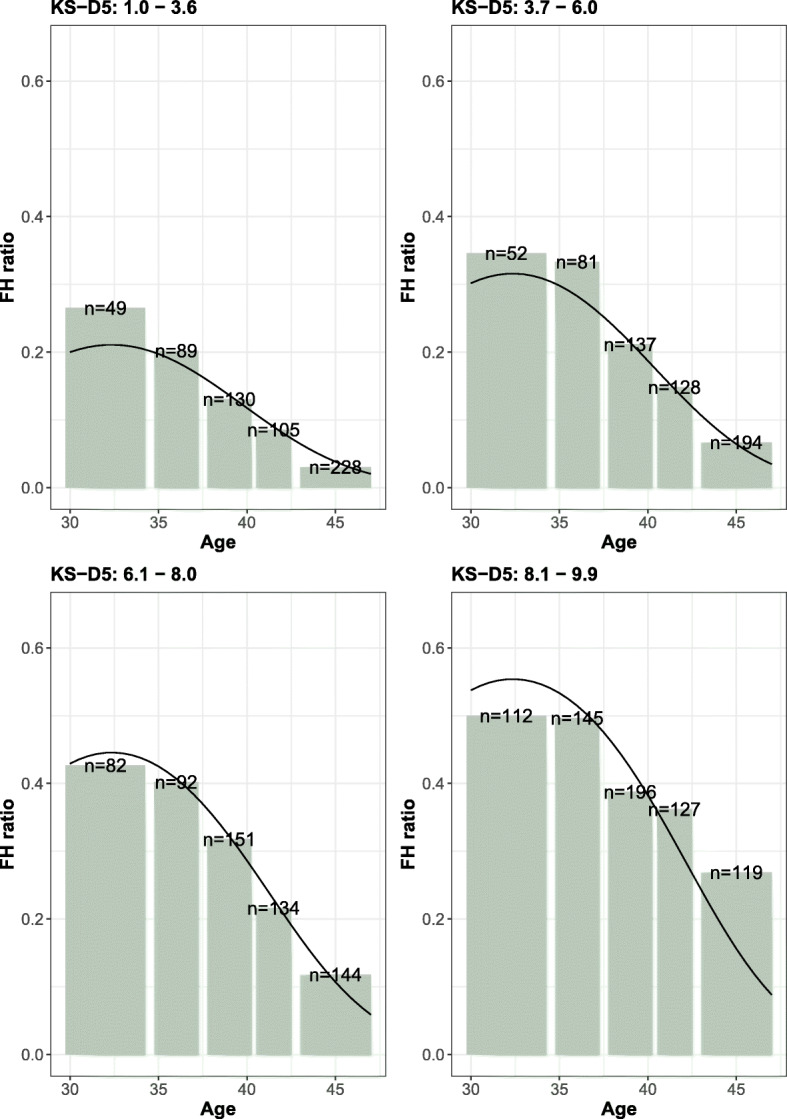
The fetal heartbeat ratio in maternal age groups. The panels show the response for embryos in different KS-D5 score subgroups. The line is the fitted multivariate logistic regression

Figure 2 shows the correlation between pregnancy rates and KS-D5 scores within the different age groups. By grouping embryos into quality groups depending on their KS-D5 scores, it is possible to investigate if the age response is affected by embryo quality. Figure 3 shows the correlation between pregnancy rates and age for the different embryo quality groups and indicates that increasing maternal age led to low pregnancy rates regardless of KS-D5 scores.

### LB+ rates stratified by maternal age group and analysis of KS-D5

LB could be analyzed in 2469 cycles. In the remaining 13 cycles, clinical outcomes data were not reported by the patients or the hospital. Table 5 shows the LB+ rates in each KS-D5 group stratified by maternal age. In the combined results from all age groups, LB+ rates significantly increased when KS-D5 scores increased (*P* <  0.05). Furthermore, within each maternal age group, LB+ rates significantly increased when KS-D5 scores increased (*P* <  0.05). In all KS-D5 groups, LB+ rates significantly decreased when maternal age increased (*P* <  0.05).

**Table 5:** LB+ rates (%) in each KS-D5 group stratified by SART maternal age

	1.0–3.6*	3.7–6.0*	6.1–8.0*	8.1–9.9*	No. of cycles	*P* value for trend test
< 35	21.4	30.2	40.5	46.4	287	< 0.05
35–37	16.7	31.3	35.8	43.1	401	< 0.05
38–40	10.6	12.0	28.1	31.8	611	< 0.05
41–42	5.9	8.7	14.9	27.8	489	< 0.05
≥ 43	0.9	4.0	5.9	19.0	681	< 0.05
Total	7.8	13.0	22.8	33.6	2469	< 0.05

*LB+* Live birth positive, *KS-D5* KIDScore Day5

* An asterisk indicates that the LB+ rates significantly decreased when maternal age increased for each column according to the Cochran-Armitage trend test (*P* < 0.05)

### Estimation of the likelihood of fetal heartbeat

Multi-variable logistic regression models were used to investigate the relationship between FHB and KS-D5 and maternal age. Due to the non-linear response to maternal age both quadratic interaction between maternal age and KS-D5 were included. In a stepwise model selection procedure, it was found that the model that best described the data included KS-D5 scores, age and age^2^ as covariates. The estimated parameters in this regression model were KS: 0.2482 ± 0.0224, age: 0.7715 ± 0.1941, age^2^: − 0.0119 ± 0.0026 and intercept: − 14.47 ± 3.63. The regression curve for the different age groups is shown in Fig. 2 and for different KS-D5 groups in Fig. 3. The equation for estimating the likelihood of fetal heartbeat is shown in supplementary Fig. 1.

### Comparison of AUCs among age groups

Table 6 shows the comparison of AUCs among maternal age groups in prediction of FHB and LB. For FHB prediction, the overall AUC of all patients was 0.680. For subgroups, the AUCs were: < 35 age group: 0.589, 35–37 age group: 0.658, 38–40 age group: 0.647, 41–42 age group: 0.673 and ≥ 43 age group: 0.737. Analyses across the groups showed that the AUC of the < 35 age group was significantly lower than that of the 41–42 and ≥ 43 age groups (*P* <  0.05). No significant difference was found among the other groups. The ROC curves for each maternal age group are shown in supplementary Fig. 2.

**Table 6:** AUCs that predicted FHB+ / LB+ after SVBT compared among all maternal age groups

	< 35	35–37	38–40	41–42	≥ 43	Total
FHB	0.589 ^a^	0.658 ^ab^	0.647 ^ab^	0.673 ^ab^	0.737 ^b^	0.680
LB	0.596 ^a^	0.640 ^a^	0.646 ^a^	0.679 ^a^	0.768 ^b^	0.681

Different subscripts are statistically significant different across the maternal age groups (*P* < 0.05)

*FHB* Fetal heart beat, *LB+* Live birth positive, *AUC* Area under the curve for prediction of FHB+ / LB+, *SVBT* Single vitrified-warmed blastocyst transfer

For LB prediction, the overall AUC of all patients was 0.681. For subgroups, the AUCs were: < 35 age group: 0.596, 35–37 age group: 0.640, 38–40 age group: 0.646, 41–42 age group: 0.679 and ≥ 43 age group: 0.768. Analyses across the groups showed that the AUC of the ≥43 age group was significantly higher than other groups (*P* <  0.05). No significant differences were found between the other groups. The ROC curves for each maternal age group are shown in supplementary Fig. 3.

## Discussion

Previous studies suggested that prediction models based on morphokinetics depend on the clinical setting [16]. This means that if a laboratory changes any embryo culture condition such as culture medium, the prediction model must at least be validated or even re-estimated. However, estimating a new prediction model requires a lot of data and also a deep insight into data science. Therefore, routine use of a clinic-specific selection model based on morphokinetics is difficult. An alternative to the use of clinic-specific models is the use of general-purpose models that have been developed based on a large volume of multi-center data. This ensures that such models work across numerous clinical settings. An example of a general-purpose embryo selection model is the KIDScore™ model. The present study showed that the KS-D5 model, version 3, was useful for predicting FHB in our clinical setting. The overall AUC of the KS-D5 model was 0.68. The AUC of a previous version of KS-D5 (version 2.0) study presented by another group under different laboratory settings was 0.60 [17], which illustrates that the KS-D5 model does work in various laboratory settings.

For FHB prediction, in terms of age groups, the AUC of the < 35 age group was below 0.60. However, for age groups > 35, the AUCs were over 0.60. Particularly, the AUCs of the ≥43 age group and the 41–42 age group were significantly higher than that of the < 35 age group. These results suggest that the specificity and sensitivity of the KIDScore prediction model are different for different maternal age groups. For LB prediction, the AUC of each age group was almost the same as the FHB prediction. After confirmed FHB, the potentially most relevant reason for miscarriage is an uterine factor [29], which would explain the close association for the AUC of FHB and LB.

To our knowledge, this is the first study performing an evaluation of a generally applicable morphokinetic model in different maternal age groups for FHB and for LB. A previous study suggested that maternal age is associated with later fertilization and slower cleavage kinetics of embryos [30, 31]. Concomitant with this, in our study tB increased when maternal age increased. Furthermore, in the ≥43 age group, the performance of KS-D5 is significant higher than in other age groups. This may reflect that the overall slower developing embryos in the older age group does in fact facilitate the differentiation of poor quality and good quality embryos by the KIDScore D5 model.

Furthermore, Maternal age correlates with embryo euploidy rates as advanced maternal age increases the risk of chromosome abnormality in oocytes [32]. Previous studies and our data suggest that the euploid embryos in younger patients would be covers a wider range of KS-D5 values, including low scores. In patients > 35 years of age, on the other hand, euploidy rates may be related to KS-D5. Unfortunately, PGT-A is not yet allowed in IVF treatment by the Japan Society of Obstetrics and Gynecology, and further correlation analyses can only be done in Japan in future studies.

We observed that when maternal age increased, KS-D5 scores decreased. A previous study suggested that advanced maternal age is associated with low blastocyst morphological grades [21], which is also shown by our study. Additionally, our study showed that tB was significantly different between age groups with low KS-D5 scores in advanced age patients. This is in line with previous studies that showed that tB is a very important factor in evaluating blastocyst quality to predict pregnancy outcomes after blastocyst transfer [33, 34]. Interestingly, one previous study suggested that morphokinetic parameters did not change with advanced maternal age before t8 [35].

We calculated estimated coefficients and odds ratios for the logistic regression between FHB+ and KS-D5 scores. The estimated coefficients in the < 35 age group resulted in a low *P* value compared with other age groups. However, this difference may be due to the low number of patients in the < 35 age group. Therefore, the model needs to be re-assessed with more data from younger patients. In fact, using age square made the model more accurate than using only single age.

In this study, we did not analyze the performance of KS-D5 in regard to the day of freezing, mainly because day 7-frozen blastocyst cases are rare. In future study, we should investigate the influence of the day of blastocyst vitrification.

It is a limitation of the study that the details of the algorithm for the KS-D5 model have not been disclosed by the manufacturer, which prevents further conclusions as we do not know how the score is calculated. Also, it should be mentioned that our clinic only used minimal stimulation and natural cycles for IVF treatment and this study used only frozen blastocyst. And this study was retrospective in nature and thus may have limitations. Therefore, in future studies, randomized controlled trials are required.

Our study has shown that it is possible to make a clinic-specific calibration between an embryo ranking model and actual pregnancy rates if maternal age is considered. However, it should be noted that an external validation should be performed before our multivariate model is applied in new clinical settings. Another important point is that the embryo ranking within a single treatment cycle as the maternal age is the same for all embryos will not change. Thus, within a treatment cycle, the highest ranked KS-D5 embryo will also be the embryo with the highest predicted pregnancy rate.

## Conclusion

In conclusion, in the present study, it was shown that KS-D5 can be used to predict FHB+ and LB+ within different maternal age groups. Therefore, the combination of KS-D5 and maternal age would enable a more accurate prediction of FHB and LB compared to using KS-D5 only. Based on the multivariate model, it is possible to estimate the likelihood of a FHB when KS-D5 scores and age are known. This will be a useful tool for patient communication prior to embryo transfer.

## Supplementary Information

**Additional file 1: Supplementary Fig. 1.** Predicted relationship between fetal heartbeat likelihood and the significant parameters in the multivariate logistic regression: KS-D5 score, age and age^2^. Note: This equation is specific to this study and should not be used in other clinics unless it is validated before use.

**Additional file 2: Supplementary Fig. 2.** Receiver operating characteristic (ROC) curves for the sorting capability of KS-D5 with regards to fetal heartbeat prediction. The line represents the ROC curve for each maternal age group.

**Additional file 3: Supplementary Fig. 3.** Receiver operating characteristic (ROC) curves for the sorting capability of KS-D5 with regards to live birth prediction. The line represents the ROC curve for each maternal age group.

## Data Availability

The data sets used and/or analyzed during the current study are available from the database of Kato Ladies Clinic on reasonable request.

## Abbreviations

aOR: Adjusted odds ratio
AUC: Area under curve
CI: Confidence interval
ER: Egg retrieval
ET: Embryo transfer
FHB: Fetal heart beat
ICM: Inner cell mass
ICSI: Intracytoplasmic sperm injection
IVF: In-vitro fertilization
KS-D5: KIDScore™ Day5
PGT-A: Pre-implantation genetic testing for aneuploidy
PN: Pronuclear
OR: Odds ratio
SD: Standard deviation
SE: Standard error
STS: Standard embryo culture system
SVBT: Single vitrified-warmed blastocyst transfer
t2: Annotations of timing to 2-cell
t3: Annotations of timing to 3-cell
t4: Annotations of timing to 4-cell
t5: Annotations of timing to 5-cell
tB: Annotations of timing to full-blastocyst
TE: Trophectoderm
tPNf: Annotations of timing to pronuclear fading

## References

[1] Gardner DK, Lane M, Stevens J, Schlenker T, Schoolcraft WB (2000). Blastocyst score affects implantation and pregnancy outcome: towards a single blastocyst transfer. Fertil Steril.

[2] Kato K, Ueno S, Yabuuchi A, Uchiyama K, Okuno T, Kobayashi T (2014). Women's age and embryo developmental speed accurately predict clinical pregnancy after single vitrified-warmed blastocyst transfer. Reprod BioMed Online.

[3] Pribenszky C, Nilselid AM, Montag M (2017). Time-lapse culture with morphokinetic embryo selection improves pregnancy and live birth chances and reduces early pregnancy loss: a meta-analysis. Reprod BioMed Online.

[4] Azzarello A, Hoest T, Mikkelsen AL (2012). The impact of pronuclei morphology and dynamicity on live birth outcome after time-lapse culture. Hum Reprod.

[5] Meseguer M, Herrero J, Tejera A, Hilligsoe KM, Ramsing NB, Remohi J (2011). The use of morphokinetics as a predictor of embryo implantation. Hum Reprod.

[6] Rubio I, Kuhlmann R, Agerholm I, Kirk J, Herrero J, Escriba MJ (2012). Limited implantation success of direct-cleaved human zygotes: a time-lapse study. Fertil Steril.

[7] Desai N, Ploskonka S, Goodman LR, Austin C, Goldberg J, Falcone T (2014). Analysis of embryo morphokinetics, multinucleation and cleavage anomalies using continuous time-lapse monitoring in blastocyst transfer cycles. Reprod Biol Endocrinol.

[8] Ezoe K, Ohata K, Morita H, Ueno S, Miki T, Okimura T (2019). Prolonged blastomere movement induced by the delay of pronuclear fading and first cell division adversely affects pregnancy outcomes after fresh embryo transfer on day 2: a time-lapse study. Reprod BioMed Online.

[9] Ohata K, Ezoe K, Miki T, Morita H, Tsuchiya R, Kaneko S (2019). Blastomere movement post first cell division correlates with embryonic compaction and subsequent blastocyst formation. Reprod Biol Endocrinol.

[10] Marcos J, Perez-Albala S, Mifsud A, Molla M, Landeras J, Meseguer M (2015). Collapse of blastocysts is strongly related to lower implantation success: a time-lapse study. Hum Reprod.

[11] Bodri D, Sugimoto T, Yao Serna J, Kawachiya S, Kato R, Matsumoto T (2016). Blastocyst collapse is not an independent predictor of reduced live birth: a time-lapse study. Fertil Steril.

[12] ESHRE Working group on Time-lapse technology (2020). Good practice recommendations for the use of time-lapse technology†. Human Reprod Open.

[13] Zaninovic N, Nohales M, Zhan Q, de Los Santos ZMJ, Sierra J, Rosenwaks Z (2019). A comparison of morphokinetic markers predicting blastocyst formation and implantation potential from two large clinical data sets. J Assist Reprod Genet.

[14] Milewski R, Kuc P, Kuczynska A, Stankiewicz B, Lukaszuk K, Kuczynski W (2015). A predictive model for blastocyst formation based on morphokinetic parameters in time-lapse monitoring of embryo development. J Assist Reprod Genet.

[15] Barrie A, Homburg R, McDowell G, Brown J, Kingsland C, Troup S (2017). Examining the efficacy of six published time-lapse imaging embryo selection algorithms to predict implantation to demonstrate the need for the development of specific, in-house morphokinetic selection algorithms. Fertil Steril.

[16] Freour T, Le Fleuter N, Lammers J, Splingart C, Reignier A, Barriere P (2015). External validation of a time-lapse prediction model. Fertil Steril.

[17] Reignier A, Girard JM, Lammers J, Chtourou S, Lefebvre T, Barriere P (2019). Performance of day 5 KIDScore morphokinetic prediction models of implantation and live birth after single blastocyst transfer. J Assist Reprod Genet.

[18] Irani M, Zaninovic N, Rosenwaks Z, Xu K (2019). Does maternal age at retrieval influence the implantation potential of euploid blastocysts?. Am J Obstet Gynecol.

[19] Demko ZP, Simon AL, McCoy RC, Petrov DA, Rabinowitz M (2016). Effects of maternal age on euploidy rates in a large cohort of embryos analyzed with 24-chromosome single-nucleotide polymorphism-based preimplantation genetic screening. Fertil Steril.

[20] Lee CI, Chen CH, Huang CC, Cheng EH, Chen HH, Ho ST (2019). Embryo morphokinetics is potentially associated with clinical outcomes of single-embryo transfers in preimplantation genetic testing for aneuploidy cycles. Reprod BioMed Online.

[21] Goto S, Kadowaki T, Tanaka S, Hashimoto H, Kokeguchi S, Shiotani M (2011). Prediction of pregnancy rate by blastocyst morphological score and age, based on 1,488 single frozen-thawed blastocyst transfer cycles. Fertil Steril.

[22] Okimura T, Kuwayama M, Segawa T, Takehara Y, Kato K, Kato O (2009). Relations between the timing of transfer, expansion size and implantation ratesin frozen thawed single blastocyst transfer. Fertil Steril.

[23] Mori C, Yabuuchi A, Ezoe K, Murata N, Takayama Y, Okimura T (2015). Hydroxypropyl cellulose as an option for supplementation of cryoprotectant solutions for embryo vitrification in human assisted reproductive technologies. Reprod BioMed Online.

[24] Ueno S, Uchiyama K, Kuroda T, Okimura T, Yabuuchi A, Kobayashi T (2020). Establishment of day 7 blastocyst freezing criteria using blastocyst diameter for single vitrified-warmed blastocyst transfer from live birth outcomes: a single-center, large cohort, retrospectively matched study. J Assist Reprod Genet.

[25] Sundvall L, Ingerslev HJ, Breth Knudsen U, Kirkegaard K (2013). Inter- and intra-observer variability of time-lapse annotations. Hum Reprod.

[26] Kato K, Takehara Y, Segawa T, Kawachiya S, Okuno T, Kobayashi T (2012). Minimal ovarian stimulation combined with elective single embryo transfer policy: age-specific results of a large, single-center, Japanese cohort. Reprod Biol Endocrinol.

[27] Ueno S, Ezoe K, Yabuuchi A, Uchiyama K, Okimura T, Okuno T (2016). Complete zona pellucida removal from vitrified-warmed human blastocysts facilitates earlier in-vitro attachment and outgrowth. Reprod BioMed Online.

[28] McLernon DJ, Steyerberg EW, Te Velde ER, Lee AJ, Bhattacharya S (2016). Predicting the chances of a live birth after one or more complete cycles of in vitro fertilisation: population based study of linked cycle data from 113 873 women. BMJ.

[29] Turocy JM, Rackow BW (2019). Uterine factor in recurrent pregnancy loss. Semin Perinatol.

[30] Akhter N, Shahab M (2017). Morphokinetic analysis of human embryo development and its relationship to the female age: a retrospective time-lapse imaging study. Cell Mol Biol (Noisy-le-grand).

[31] Dal Canto M, Bartolacci A, Turchi D, Pignataro D, Lain M, De Ponti E (2021). Faster fertilization and cleavage kinetics reflect competence to achieve a live birth after intracytoplasmic sperm injection, but this association fades with maternal age. Fertil Steril.

[32] Hassold T, Hunt P (2001). To err (meiotically) is human: the genesis of human aneuploidy. Nat Rev Genet.

[33] Storr A, Venetis CA, Cooke S, Susetio D, Kilani S, Ledger W (2015). Morphokinetic parameters using time-lapse technology and day 5 embryo quality: a prospective cohort study. J Assist Reprod Genet.

[34] Kirkegaard K, Kesmodel US, Hindkjaer JJ, Ingerslev HJ (2013). Time-lapse parameters as predictors of blastocyst development and pregnancy outcome in embryos from good prognosis patients: a prospective cohort study. Hum Reprod.

[35] Warshaviak M, Kalma Y, Carmon A, Samara N, Dviri M, Azem F (2019). The effect of advanced maternal age on embryo Morphokinetics. Front Endocrinol (Lausanne).

