# Supplementary figures and images for: Comparing prediction of ongoing pregnancy and live birth outcomes in patients with advanced and younger maternal age patients using KIDScore™ day 5: a large-cohort retrospective study with single vitrified-warmed blastocyst transfer
Source: Reprod Biol Endocrinol. 2021 Jul 2;19:98. doi: 10.1186/s12958-021-00767-4 (PMC8252298; doi:10.1186/s12958-021-00767-4)

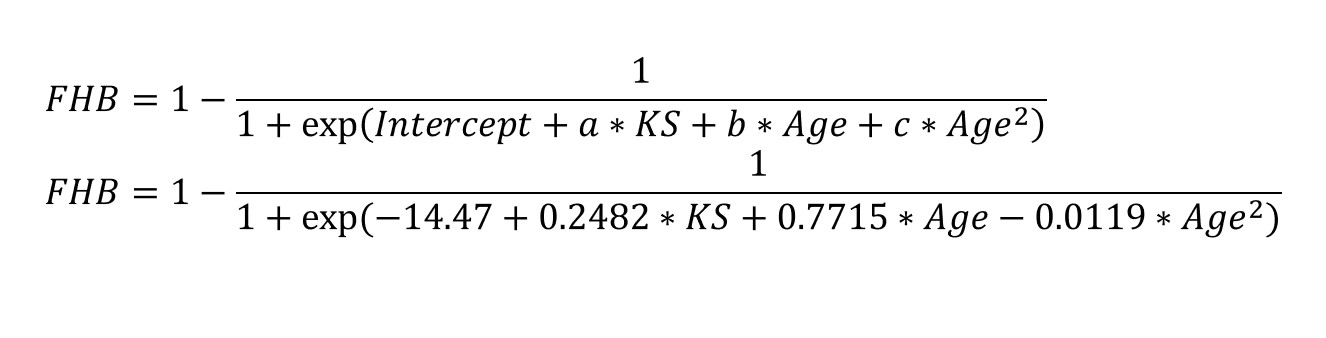

Supplement: Supplementary file 1 — Additional file 1: Supplementary Fig. 1. Predicted relationship between fetal heartbeat likelihood and the significant parameters in the multivariate logistic regression: KS-D5 score, age and age2. Note: This equation is specific to this study and should not be used in other clinics unless it is validated before use. [file 12958_2021_767_MOESM1_ESM.jpg]

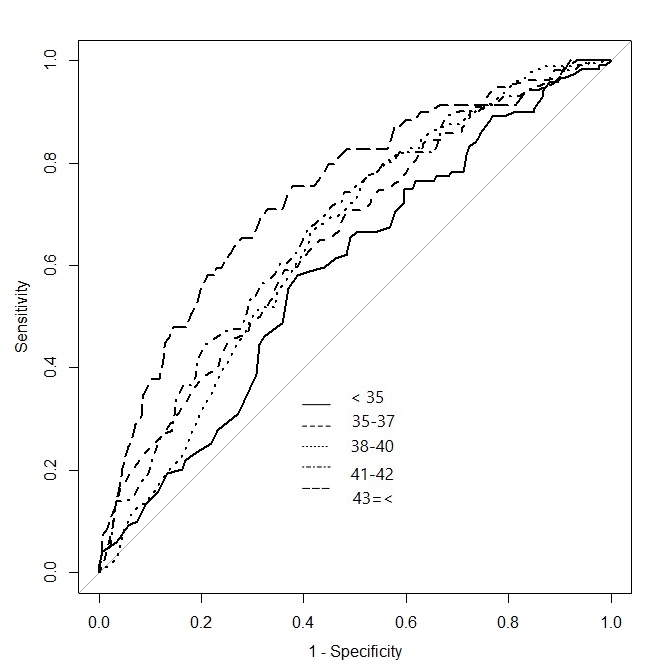

Supplement: Supplementary file 2 — Additional file 2: Supplementary Fig. 2. Receiver operating characteristic (ROC) curves for the sorting capability of KS-D5 with regards to fetal heartbeat prediction. The line represents the ROC curve for each maternal age group. [file 12958_2021_767_MOESM2_ESM.jpeg]

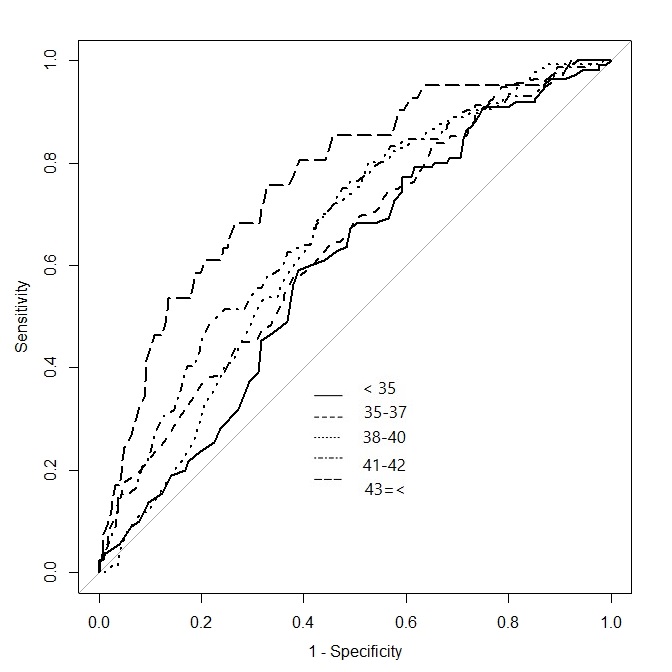

Supplement: Supplementary file 3 — Additional file 3: Supplementary Fig. 3. Receiver operating characteristic (ROC) curves for the sorting capability of KS-D5 with regards to live birth prediction. The line represents the ROC curve for each maternal age group. [file 12958_2021_767_MOESM3_ESM.jpeg]
